# The Use of Flow-Injection Analysis with Chemiluminescence Detection of Aqueous Ferrous Iron in Waters Containing High Concentrations of Organic Compounds

**DOI:** 10.3390/s90604390

**Published:** 2009-06-04

**Authors:** Christopher J. Borman, B. Patrick Sullivan, Carrick M. Eggleston, Patricia J. S. Colberg

**Affiliations:** 1 Department of Chemistry, University of Wyoming, Dept 3838, 1000 E. University Avenue, Laramie, Wyoming 82071, USA; 2 Department of Geology and Geophysics, University of Wyoming, Dept 3006, 1000 E. University Avenue, Laramie, Wyoming 82071, USA; E-Mail: carrick@uwyo.edu (C.M.E.); 3 Department of Civil and Architectural Engineering, University of Wyoming, Dept 3295, 1000 E. University Avenue, Laramie, Wyoming 82071, USA; E-Mail: pczoo@uwyo.edu (P.J.S.C.)

**Keywords:** flow-injection analysis, chemiluminescence, ferrous iron determination, iron oxide dissolution, luminol, Felume

## Abstract

An evaluation of flow-injection analysis with chemiluminescence detection (FIA-CL) to quantify Fe^2+^_(aq)_ in freshwaters was performed. Iron-coordinating and/or iron-reducing compounds, dissolved organic matter (DOM), and samples from two natural water systems were used to amend standard solutions of Fe^2+^_(aq)_. Slopes of the response curves from ferrous iron standards (1 – 100 nM) were compared to the response curves of iron standards containing the amendments. Results suggest that FIA-CL is not suitable for systems containing ascorbate, hydroxylamine, cysteine or DOM. Little or no change in sensitivity occurred in solutions of oxalate and glycine or in natural waters with little organic matter.

## 1. Introduction

Quantitative analysis of metal ions in natural waters is essential to understanding global biogeochemical cycling. The study of iron species and compounds in aqueous solution has been of particular interest over the past 30 years due to their role in various chemical, physical and biological processes in natural waters. These processes are important in understanding the biogeochemistry of iron and the impacts of contaminants on the systems. Iron (hydr)oxides have been shown to act as sorbents of organic and other metal species [1,2]. Iron is an essential nutrient [3-5], and is abundant in many mineral oxides that exchange iron with surface and ground waters through dissolution and precipitation [6-10]. Iron is often the most abundant redox-active metal ion in natural waters and is crucial to biota during electron transport [5,11-14]. The oxidation state and speciation of iron dictates its bioavailability, and may directly affect both the toxicity and availability of many chemicals in the environment.

Numerous analytical techniques for determining aqueous iron concentration have been developed and deployed [15], including titrimetric [16], electrochemical [17-19], chromatographic [20], ultracentrifugation [21], and photometric methods [22-24]. Flow-injection analysis (FIA) techniques have facilitated simple, accurate and precise determinations when coupled with spectrophotometric detection of chromophoric metal complexes but, there are some limitations of colorimetric analyses that result in inaccurate determinations [15,25,26]. Moreover, spectrophotometric determination of sub-micromolar analyte concentrations (e.g. Fe^2+^_(aq)_) is often difficult without extending optical pathlength or pre-concentrating analytes in samples [22,27,28]. One solution for these limitations is the use of chemiluminescence (CL) detection coupled with FIA, which has been shown to be rapid and highly sensitive in the quantitation of aqueous ferrous iron [29-36].

### 1.1. Chemiluminescence of Luminol

**Scheme 1.**
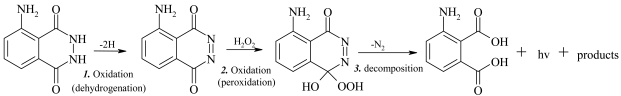
The general reaction of luminol to produce light for determination of aqueous Fe(II). Fe^2+^_(aq)_ ‘catalyzes’ the second step in this reaction scheme. The light emitted after the third step proportional to [Fe^2+^_(aq)_] within a certain concentration range and is subject to changes in sensitivity that depend on the presence of species that can complex Fe^2+^_(aq)_ and/or impact the generation of H_2_O_2_.

Many compounds have been shown to emit light upon oxidation; the most studied is probably 5-amino-2,3-dihydro-1,4-phthalazinedione, commonly known as luminol. Albrecht (1928) [37] was the first to report the strong CL of luminol and its derivatives during their oxidation in basic solutions. Oxidants like hypochlorites and ferricyanides in association with H_2_O_2_ produced the greatest CL in early work [37,38], but since then many other species have been identified that degrade luminol with concomitant light emission [30,34,39-42]. Many ions (e.g., Fe^2+^) and compounds that are able to form reactive oxygen species (ROS) in aqueous solution are potentially suitable for determination by flow-injection analysis by chemiluminescence detection (FIA-CL) using luminol. In Scheme 1, aqueous Fe^2+^ ‘catalyzes’ the second step of the reaction scheme [34]. Actually, Fe^2+^ is oxidized, and is therefore not a true catalyst, however it is a bystander in the chemiluminescence of luminol here.

An FIA-CL instrument has been developed (FeLume – Waterville Analytical, Waterville, ME) that may be configured to determine several analytes (Co^2+^, Cu^2+^, Fe^2+^, Cr^2+^, NO_3_^-^, PO_4_^3-^ and H_2_O_2_). The FeLume has been used specifically to determine sub-nanomolar concentrations of ferrous iron in both marine and freshwaters [30,42,43].

When an iron-containing sample is free of organic matter, the relationship between chemiluminescence and [Fe_(aq)_^2+^] is approximately linear between 1 – 1,000 nM Fe_(aq)_^2+^. In the presence of fulvic acid, the linear dynamic range (LDR) is reduced to 1 – 32 nM [34]. It has been suggested that FIA-CL analysis of freshwater samples will not work well due to interferences by dissolved organic carbon (DOC) [44], while in coastal seawater, O'Sullivan *et al.* [32] found that DOM reduced the sensitivity of FIA-CL analysis of Fe(II). Similar results were obtained by Ussher *et al.* [36] in their evaluation of the effect of model ligands on Fe(II) analysis in seawater. Recently, researchers demonstrated other potential interferences that may occur with redox-active metals that produce CL of luminol [45] or species that interfere with the peroxy-luminol reaction leading to CL (step 2 in Scheme 1) [46].

Coordination of Fe^2+^ by organic chelators and low pH both contribute to stabilizing iron against oxidation by O_2_ [47-49]. Such stabilization may either depress or enhance the CL and resultant signal returned by the FeLume. Tight coordination of Fe^2+^ by organic species that persist in the mixing chamber of the FeLume results in lowering of the signal due to slower formation of the ROS required for CL of luminol. Low pH may produce a higher signal by slowing pre-injection oxidation of Fe^2+^_(aq)_, yielding more H_2_O_2_ in the mixing chamber. Species that have strong affinity for ROS like ascorbate also act to suppress the CL of luminol by scavenging radicals necessary for step 2 in the mechanism shown in Scheme 1.

**Figure 1.**
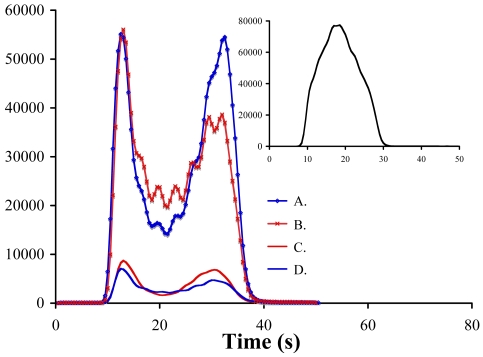
Injection peaks showing typical response generated from nanomolar [Fe^2+^] in this work. A. 100 nM Fe^2+^; B. 100 nM Fe^2+^ with 1 mg C L^-1^ fulvic acid (FA); C. 10 nM Fe^2+^; D. 10 nM Fe^2+^ with 1 mg C L^-1^ FA. Inset: Typical Gaussian response curve generated from injection of higher concentration Fe^2+^ (44.64 mM) than used in this study. The higher concentration apparently produces enough O_2_^-^ to overcome the effect low pH has on the signal.

Typical injection peaks (Figure 1) from this work demonstrate that certain organic compounds reduce the sensitivity of ferrous iron quantitation. The “doublet” peak shown in Figure 1 (typical at nanomolar [Fe^2+^]) is due to the acid in the samples overcoming the buffer capacity of the luminol solution. Lower pH decreases the signal by reducing luminol dehydrogenation (step 1 in the mechanism in Scheme 1) at nanomolar [Fe^2+^], but otherwise does not alter the relationship between signal and [Fe^2+^]. The changes in FeLume response we observed in our initial work with determination of [Fe^2+^] in the presence of organic matter served as motivation for the present study in which we systematically evaluated the performance of the FeLume in analysis of both natural and model water systems containing Fe(II) and organic species.

## 2. Experimental

### 2.1. Reagents and Samples

All chemicals (except H_2_SO_4_) were reagent grade. Oxalic acid dihydrate and ferrous ammonium sulfate hexahydrate were purchased from J.T. Baker Chemical Co. (Phillipsburg, NJ, USA). L-Ascorbic acid, glycine, and hydroxylamine hydrochloride were supplied by Fisher Scientific (Fair Lawn, NJ, USA). Hydrazine dihydrochloride and L-cysteine were purchased from Sigma-Aldrich (St. Louis, MO, USA). Sulfuric acid, veritas, redistilled was acquired from GFS Chemicals (Columbus, OH, USA). Suwannee River humic and fulvic acid standards were purchased from the International Humic Substance Society (IHSS. St. Paul, MN, USA).

Natural water samples collected from a mountain stream (Middle Crow Creek) and an unnamed alpine lake, both in SE Wyoming, USA, were acidified to pH 3, stored in the dark at room temperature, and analyzed within three weeks of collection. Middle Crow Creek is an undeveloped watershed at about 2,400 m elevation that originates near Pole Mountain in the Laramie Range in SE Wyoming, USA. This area is impacted by livestock grazing, nearby motorized traffic and human recreation (fishing and hiking). There is significant input of organic matter from overhanging trees and streamside bushes. Our research group has studied the site for several years.

The small alpine lake is located in the Snowy Range of SE Wyoming at ∼ 3,300 m above sea level. At this elevation there is little organic input from trees and shrubs, but there are grasses and other vegetation along the lakeshore. Human impact on this lake is limited to nearby camping and hiking; there are no anglers, and cattle are excluded from the area.

All solutions were prepared with 18.2 MΩ Millipore reverse-osmosis, de-ionized (RO) water and H_2_SO_4_. All samples were acidified to pH 3 [50], which slows the oxidation of ferrous iron [32,51,52]. Iron standards, including those used in experiments with various organic amendments, were made by serial dilution of a 400 μM stock solution of ferrous ammonium sulfate hexahydrate (Fe(NH_4_)_2_(SO_4_)_2_·6H_2_O) in 0.1 M H_2_SO_4_. The concentrations of organic amendments (10^-2^ M, 10^-4^ M and 10^-6^ M) are similar to those used in dissolution and reduction experiments involving iron (hydr)oxide minerals and colloids [7,10,53-55], and organic carbon concentrations for the humic and fulvic acid experiments were those typical of surface fresh waters [56] and were added for final concentrations of 1, 5, or 10 mg C L^-1^.

Ferrous iron was added in standard additions (1 – 100 nM) to samples amended with select organic compounds or containing natural organic matter (NOM). Certain organic species are used in exploring the biogeochemistry of iron because they promote the reductive dissolution of iron and so are models for natural reductive dissolution processes. We have selected compounds that are involved in the coordination of iron and/or reducing Fe(III) centers at water-mineral interfaces. Response curves generated from Fe^2+^-spiked experimental samples measured by FIA-CL (FeLume) were directly compared to the response of Fe^2+^ standards in RO water at pH 3. The experimental samples were made from five commonly used iron-complexing and iron-reducing species at three concentrations in pH 3 RO water or in water samples collected from a mountain stream and an alpine lake as described above.

### 2.2. Instrumentation and Glassware

All glassware was cleaned thoroughly, sequentially acid (10% v_HCl_/v_H2O_) and base washed (0.1 M NaOH or KOH) for a minimum of 24 hours, rinsed thoroughly with reverse-osmosis (RO) water, and air-dried overnight. The FeLume was used as configured by the manufacturer for ferrous iron determination. The instrument was interfaced with a PC running LabView v.6.1 (National Instruments Corp., Austin, TX, USA) with a USA-49W USB 4-port serial adaptor (Keyspan, Richmond, CA, USA). A Dynamax RP-1 peristaltic pump (Rainin Instruments, Oakland, CA, USA) delivered (at 20 rpm) the carrier, luminol, and sample solutions through Tygon (Cole Parmer Instrument Co., Vernon Hills, IL, USA) and Teflon FEP tubing (Upchurch Scientific, Inc., Oak Harbor, WA, USA) to the reaction chamber of the FeLume. Samples were injected by a 0.1 mM Na_2_CO_3_ carrier solution where they mixed with a continuous flowing buffered solution (NH_3_/NH_4_Cl at pH 10) of luminol (0.5 mM). Upon mixing, Fe(II) is rapidly (milliseconds) oxidized forming the reactive oxygen species (ROS), superoxide (O_2_^-^), in the solution, which catalyzes the multi-step oxidation of luminol that produces 426 nm light (see Scheme 1 above) This light is detected using a Hamamatsu HC135 photon counter (PMT) whose output voltage is proportional to [Fe_(aq)_^2+^]. The integral of the PMT signal plotted versus Fe(II) concentration of standards provided linear plots in the range of 1 to 100 nM Fe^2+^.

Each data point is the average of five replicates and as such error bars on plots denote 95% confidence limits (*tsn^-2^*), where *t* is from student's t-table, *s* is the standard deviation of the five measurements, and *n* is the number of measurements. The slope of the linear response curve of each experiment was then divided by the slope of the ferrous iron calibration curve, providing normalized slope (*m_N_*) that was used as a parameter of effectiveness of the analytical method (Tables 1 and 2). Each experiment was run twice to yield an average *m_N_* for each experimental system.

## 3. Results and Discussion

The FeLume exhibited slight differences in signal response from day to day that may be attributed to luminol aging, pump tube stretching, or variation in PMT output. Since we did not optimize the signal-to-noise ratio before each experiment [42,50], standard calibration response curves were collected for each individual experiment. Linear calibration curves were generated for comparison purposes only, and were not used to calculate [Fe^2+^]_(aq)_ in the amended samples. These comparisons enabled us to evaluate the extent of any interference by organic species in such iron determinations.

### 3.1. Chelators and Reductants

Ascorbate and oxalate, both separately and in tandem, are routinely used in mineral dissolution kinetic studies both alone and in combination at concentrations ranging from 10^-5^ to 10^-3^ M [7,10,55]. Cysteine and hydroxylamine hydrochloride have been used in similar studies at 10^-2^ M [53,54]. Glycine was included in this study as a comparison to cysteine, which was used by Seitz and Hercules to assess the effect on coordination on the CL of luminol [41]. We also included another commonly used reductant, hydrazine, because it is a reagent we use in a spectrophotometric method for total aqueous iron determination [57].

#### 3.1.1. Ascorbate and oxalate

**Figure 3.**
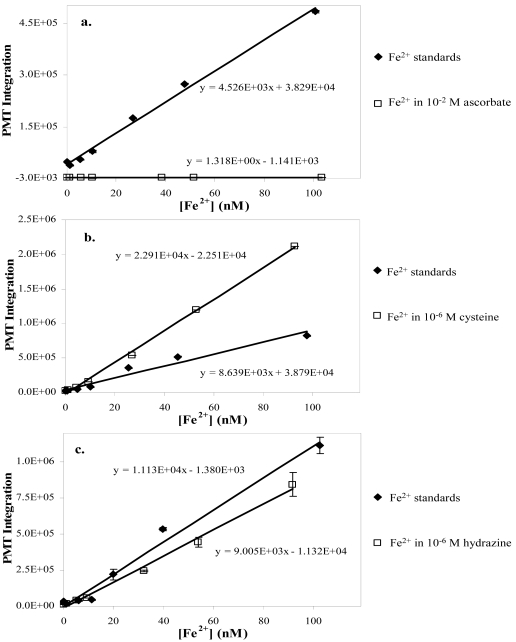
Three FeLume response comparisons (vs. Fe^2+^ only standards) of selected organic compounds demonstrating: a). signal quenching of 10^-2^ M ascorbate (negative integration values represent artifact due to subtraction from baseline); b). signal enhancement of 10^-6^ M cysteine; and c). minimal signal quenching of 10^-6^ M hydrazine.

Though ascorbate has been reported to enhance the CL of luminol in at least one system [58], at all of the concentrations that we evaluated ascorbate quenched the CL of luminol (10^-2^ M ascorbate plotted in Figure 3a).

The response was near zero across the entire range of Fe^2+^ concentrations (1 – 100 nM) and produced normalized slopes that were negative or very small (ca. 10^-4^). The effect of oxalate addition on the FeLume response was negligible at oxalate concentrations of 10^-4^ and 10^-6^ M (data in Table 1) with *m_N_* values near unity. At 10^-2^ M oxalate, however, the signal response was double the standard response. In the combined ascorbate/oxalate system, the quenching effect of ascorbate on CL apparently limited the sensitivity at 10^-2^ M and 10^-4^ M by producing a flat response like that observed with the ascorbate alone (see Figure 3a). At 10^-6^ M ascorbate/oxalate, the response was only slightly diminished (∼ 0.64), which should not be problematic for [Fe^2+^] determinations.

#### 3.1.2. Cysteine and glycine

Cysteine has been reported to enhance the CL of luminol even though a decreased luminol CL signal usually results when antioxidants are injected into an oxidant stream [58]. Wheatley and coworkers saw an increase in the CL of luminol [40]; however, the reductant they used was cobalt(II) rather than iron(II), which may account for the observed difference from our results. At 10^-2^ M cysteine, the FeLume response was near zero throughout the 1 – 100 nM Fe^2+^ range, making this analytical method useless at high cysteine concentrations. The slope of the line from the 10^-4^ M cysteine experiment approached 40% of the standard response, suggesting ferrous iron determination is possible in solutions at that cysteine concentration. The lowest cysteine concentration at 10^-6^ M, exhibited a dramatic enhancement in response with an *m_N_* of ∼ 2.6 (Figure 3b).

Although Seitz and Hercules [41] reported a decrease in CL as a function of glycine concentration, glycine at 10^-2^ M and 10^-4^ M had little effect on the FeLume response in our experiments; in fact, glycine enhanced the FeLume response at 10^-6^ M (see Table 1). The only difference in their work appears to be in their buffer – KOH/H_3_BO_4_ versus NH_3_/NH_4_Cl used here. Our data suggest that glycine does not adversely affect Fe^2+^ determinations using the FeLume.

#### 3.1.3. Hydroxylamine and hydrazine

Hydroxylamine at 10^-2^ M had a flat response curve (not CL quenching) with a significant signal that remained relatively unchanged throughout the [Fe^2+^] range. The experiments at 10^-4^ and 10^-6^ M produced *m_N_* values between 0.40 – 0.45, representing a slight attenuation in CL response that would not make for inaccurate [Fe^2+^] determinations. The effect hydrazine had on the FeLume response appears to be a direct function of concentration. At the highest concentration, the signal was greatly attenuated (*m_N_* = 0.082), while at 10^-4^ and 10^-6^ M (Figure 3c), the FeLume exhibited slight reduction in sensitivity with *m_N_* values of 0.25 and 0.75, respectively (Table 1).

### 3.2. Natural Waters, Humic and Fulvic Acids

We assessed the effectiveness of the FeLume in the analysis of both natural waters and model water systems (Table 2), all of which contained or were amended with DOM, humic and/or fulvic acids. The samples from Middle Crow Creek (MCC) and the small alpine lake (SR lake) contained between 5 and 10 mg C L^-1^ DOM and had aqueous Fe^2+^ concentrations that ranged from 1 to 8 μM.

The concentrations of DOM in our experimental solutions were chosen based on published values for carbon concentration (mg C L^-1^) [46]. We chose three concentrations of carbon amendments (1, 5 and 10 mg C L^-1^) and performed the analyses and comparisons as were done for the organic ligands.

#### 3.2.1. Humic and fulvic acids

**Figure 4.**
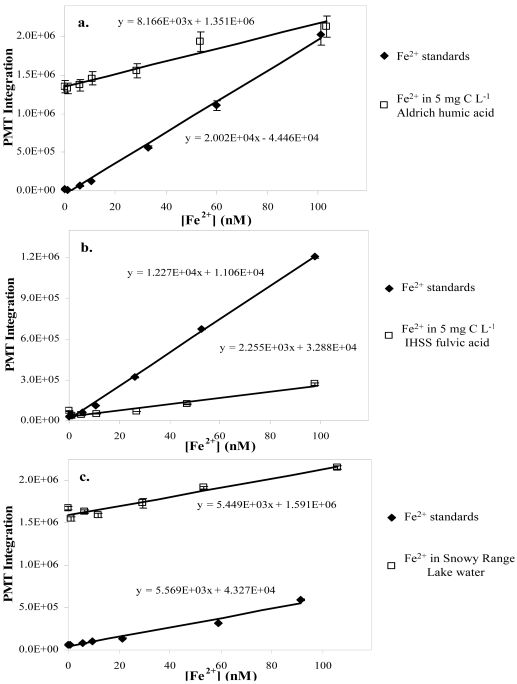
Three FeLume response comparisons (vs. Fe^2+^ standards) of natural water and natural organic matter: a). Aldrich humic acid; b). IHSS fulvic acid c). sample from SR lake.

In the experiment using Aldrich humic acid (lot # 03130JS), we found that at 5 and 10 mg C L^-1^ the CL response was attenuated with *m_N_* values between 0.35 – 0.37 (Figure 4a). O'Sullivan *et al.* [32] reported a similar reduction in sensitivity in waters containing high DOC.

Our experiments with this lot of humic acid were probably less sensitive to increasing [Fe^2+^] because of its high iron content (7,700 mg L^-1^), resulting in an elevated signal of the blank, while the flat response indicated interference by the humic material. The solution containing the least humic acid (1 mg C L^-1^) also exhibited reduced sensitivity (*m_N_* ∼ 0.7), but still allowed [Fe^2+^] determination.

The IHSS humic acid (HA) and fulvic acid (FA) systems exhibited behaviors similar to the Aldrich HA just discussed (Figure 4b). In both HA and FA experiments, the two higher concentrations (5 and 10 mg C L^-1^) had lower sensitivities (*m_N_* ∼ 0.17 to 0.19, respectively) than those of Aldrich HA. Both response curves had y-intercepts much closer to zero than the corresponding Aldrich HA curves, indicating lower iron content in the much purer IHSS acids. The smaller normalized slopes (*m_N_*) are likely due to greater complexation of aqueous Fe^2+^. Complexation of Fe^2+^ with DOM should lower its reactivity and decrease the iron-catalyzed generation of ROS needed for CL of the luminol in this system. The response of both humic and fulvic acid systems at 1 mg C L^-1^ is only slightly depressed (*m_N_* values of 0.77 and 0.83), indicating that at low concentrations this analytical method is still useful.

#### 3.2.2. Natural water samples

The natural water samples exhibited divergent results. The response of the MCC samples appeared negative with respect to iron concentration. High iron concentrations in the MCC sample with the addition of Fe^2+^ spikes exceeded the LDR of the method, thus making this analytical tool incapable of [Fe^2+^] determination without dilution of the sample. The FeLume exhibits non-linear (sigmoidal) behavior as the PMT nears its over-voltage limit. The SR lake sample (Figure 4c) had a positive response with added ferrous iron (*m_N_* of 0.86), suggesting this technique is useful for analysis at this site. Analyses of waters at both sites indicate high aqueous iron and total organic carbon (TOC); the high iron concentrations explain the elevated blank signal for both the lake and creek response curves, but with similar TOC content the divergent responses are difficult to explain without further analysis of the systems. There may be a difference in the type or quality of organic matter input, leading to variable coordination affinity for iron or perhaps different radical scavenging ability.

### 3.3. Method Evaluation

In determining the effectiveness of the FeLume technique for Fe^2+^ analysis in natural waters and in systems containing organic compounds, *m_N_* values of 0.9 or greater were those we considered fully effective; *m_N_* values between 0.1 and 0.9 were still effective but at reduced sensitivity. Values less than 0.1 are not recommended for use without verifying the sensitivity as appropriate for the iron concentration expected.

The CL quenching seen in the ascorbate systems may be due to competition for radicals by ascorbate [59,60], but iron coordination by ascorbate probably contributes by reducing CL through ligand stabilization of Fe(II) against oxidation. As reported previously, iron-coordinating species appear to interfere with the iron-catalyzed CL of luminol [30,32,41]. Coordination of iron by ascorbate - or more complex organic matter - stabilizes it against oxidation by dissolved oxygen and subsequent formation of ROS required for the production of CL from luminol [30,32,47,49].

Ascorbate is such an effective quencher of luminol CL by ferrous iron that, at 10^-2^ and 10^-4^ M in the combined ascorbate/oxalate system, any signal enhancement due to oxalate is overcome; however, at low concentrations (i.e., 10^-6^ M) of both coordinator and reductant, the response appears to be manageable. At these concentrations, *m_N_* is depressed only ∼0.3 from the oxalate system, suggesting dissolution experiments of iron oxides involving ascorbate and oxalate might be feasible. Determination of [Fe^2+^] by the FeLume is mostly unencumbered in solutions containing oxalate up to 10^-4^ M; in fact, this method appears to be highly sensitive in oxalate solutions of 10^-2^ M. Signal enhancement may be due to slight pH lowering of the sample solution by oxalate amendment further stabilizing Fe^2+^ against oxidation prior to injection - higher [Fe^2+^] result in greater CL from the mixing chamber.

Discussions of mechanisms in the augmentation of signal by cysteine is speculative, but others have reported CL of luminol by cysteine [58] and thus our results may reflect a simple additive effect. It is possible that oxidation of cysteine by dissolved oxygen may generate superoxide. This alternate source of superoxide - one of the ROS associated with the reaction that generates CL of luminol - is likely to enhance the signal. Regardless of the mechanism of signal enhancement, the FeLume is an effective tool for measuring [Fe^2+^] in solutions containing cysteine concentrations up to 10^-4^ M, but should be avoided at higher concentrations, as samples containing significant cysteine appear to result in anomalously high [Fe^2+^] by this method.

Previous reports of depressed CL of luminol in the presence of glycine [41] might be attributed to the difference in buffer types; however, further study is required to verify this discrepancy. With the exception of glycine at 10^-4^ M (*m_N_* ∼ 0.87), there appears to be an enhancement in the CL response by this amino acid. This may be attributed to glycine amendment pH lowering as discussed with oxalate above. Since the normalized slope for the experiment at 10^-4^ M glycine is only slightly less than our somewhat arbitrary cutoff for *m_N_* (0.90), this appears to be an effective method in the presence of glycine at these concentrations.

The normalized slopes determined from the experiments containing the reductants hydroxylamine and hydrazine up to 10^-4^ M suggest the FeLume is suitable for Fe^2+^ determination. Hydroxylamine and hydrazine at 10^-2^ M concentration quench the CL of luminol to such an extent that [Fe^2+^] quantitation is impractical or impossible.

Care must be taken in measuring Fe^2+^_(aq)_ with the FeLume in samples with potentially high levels of iron complexed to NOM, as iron tends to dissociate over time leading to higher “free” iron concentrations. Often DOM interferes with CL of luminol by Fe^2+^, but sometimes the presence of DOM does not affect the sensitivity of the method. The natural water sample from the alpine lake contains significant iron, causing an offset in response (large positive y-intercept), but the experimental response curve is nearly parallel to that of the calibration curve indicating little effect on method sensitivity. The samples from MCC, however, quench CL to the point that there is no correlation between added iron and the instrumental response. At the lower concentration range typical of natural surface waters (less than 5 mg C L^-1^), humic and fulvic acids may slightly depress the sensitivity of the method, but do not preclude its use.

### 3.4. Recommendations

The results of experiments with ascorbate indicate that it renders the method ineffectual at the concentrations used. Ascorbate is considered such an effective iron chelator and reductant of iron (hydr)oxides [7,10,55] that lower concentrations may be used in model dissolution studies and should be tested for interference with the FeLume response at those concentrations. Oxalate either does not dramatically affect the response or enhances its sensitivity (seen at high concentrations), and we recommend its use with the FeLume during ferrous iron analyses. Cysteine may be used at ∼ 10^-6^ M with the FeLume, but starts to diminish in sensitivity as the concentration increases to 10^-4^ M or above. We recommend this method in systems containing glycine at concentrations between 10^-6^ and 10^-2^ M using the NH_3_/NH_4_Cl buffer system, as it appears to not affect the CL of luminol by Fe^2+^. The results for these two amino acids suggest pursuing similar studies with other amino acids capable of reductive dissolution of iron minerals. The reductants hydroxylamine and hydrazine may be used at concentrations in the range of 10^-6^ M, but like cysteine, they start to reduce the CL signal at higher concentrations.

Measurements of ferrous iron in samples containing high DOM concentrations are generally not facile and should be avoided. We discourage the direct use of the FeLume on samples where high concentrations of iron may be coordinated with DOM - in such systems colorimetry may be sufficient. Middle Crow Creek water, and samples containing Aldrich HA at similar carbon content, both had such high levels of iron and DOM that this analytical method was rendered ineffective. Samples containing IHSS HA and FA up to 10 mg C L^-1^ and Aldrich HA up to 5 mg C L^-1^ can be effectively analyzed for ferrous iron by the FeLume. Other, more pristine natural water sites have water suitable for Fe^2+^ quantitation using the FeLume, as our site in the Snowy Range showed, but its effectiveness should be determined on a site-by-site basis. All samples containing Fe(III)-organic complexes (especially oxalate) should be kept dark prior and during analysis to ensure photoreduction of these complexes does not result in altering [Fe^2+^] determination [35,61-65].

In all cases in the determination of [Fe^2+^], the response of the FeLume should be checked by comparing iron-spiked samples with iron standards. This will provide a baseline response and indicate whether the FeLume needs signal-to-noise optimization by fine-tuning the PMT voltage or by varying luminol concentration.

## Figures and Tables

**Figure 2.**
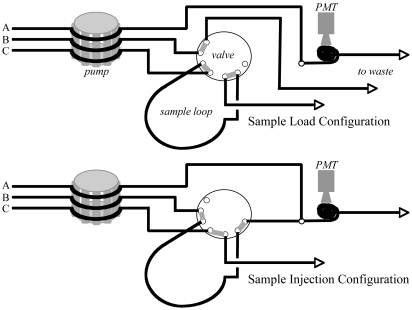
FeLume sample loading and injection configurations. In both configurations, the luminol (line A) runs directly to the mixing chamber. In the 90-second sample loading configuration (top), the carrier solution (line B) runs to waste, and the sample (line C) charges the loop (pre-injection). In the injection configuration (50 s.), the carrier solution runs through the sample loop, rapidly moving the sample into the mixing chamber where the resulting CL is detected by the PMT (all arrows run to waste container).

**Table 1.** Organic chelators and reductants.

**Amendment**	**Concentration**	**m_N_^a^**	**Effectiveness**
Ascorbate	10^-6^ M	< 0.1	NR
	10^-4^ M	< 0.1	NR
	10^-2^ M	< 0.1	NR
Oxalate	10^-6^ M	0.9(0)	Yes
	10^-4^ M	1.1	Yes
	10^-2^ M	2.(0)	Yes
Ascorbate/oxalate	10^-6^ M	0.6(4)	LS
	10^-4^ M	< 0.1	NR
	10^-2^ M	< 0.1	NR
Cysteine	10^-6^ M	2.(6)	Yes
	10^-4^ M	0.3(7)	LS
	10^-2^ M	< 0.1	NR
Glycine	10^-6^ M	1.(8)	Yes
	10^-4^ M	0.8(8)	LS
	10^-2^ M	1.3	Yes
Hydroxylamine	10^-6^ M	0.4(5)	LS
	10^-4^ M	0.4(2)	LS
	10^-2^ M	< 0.1	NR
Hydrazine	10^-6^ M	0.7(5)	LS
	10^-4^ M	0.2(5)	LS
	10^-2^ M	< 0.1	NR

Notes: a – ± 6.4 %; NR – not recommended; LS – lowered sensitivity

**Table 2.** Dissolved organic matter and natural water samples.*

**Amendment**	**Concentration**	**m_N_^a^**	**Effectiveness**
Aldrich Humic Acid	1 mg C L^-1^	0.7(0)	LS
	5 mg C L^-1^	0.3(6)	LS
	10 mg C L^-1^	0.3(7)	LS
IHSS Humic Acid	1 mg C L^-1^	0.8(4)	LS
	5 mg C L^-1^	0.1(7)	LS
	10 mg C L^-1^	0.1(9)	LS
IHSS Fulvic Acid	1 mg C L^-1^	0.7(7)	LS
	5 mg C L^-1^	0.1(9)	LS
	10 mg C L^-1^	0.1(7)	LS
MCC water	∼ 5 mg C L^-1^*	<0.1	NR
SR lake water	∼ 10 mg C L^-1^*	0.8(6)	LS

Notes: a – ± 6.4 %; NR – not recommended; LS – lowered sensitivity;

* Natural waters organic carbon content measured previously by TOC analysis.
